# Correction: How could simulations elucidate Na_v_1.5 channel blockers mechanism?

**DOI:** 10.1085/jgp.20241373001082025c

**Published:** 2025-01-16

**Authors:** Tanadet Pipatpolkai

Vol. 157, No. 2 | https://doi.org/10.1085/jgp.202413730 | January 07, 2025

In the original commentary, the wording was misleading regarding the location of the drug binding sites versus the route of access discussed in the related article by Tao and Corry (2025; https://doi.org/10.1085/jgp.202413658). The commentary incorrectly stated that drugs that bind in the center of the pore “enter from the cytoplasm” and labeled these cytoplasmic blockers; in fact, all of the compounds discussed in the article are known tonic blockers, meaning they do not need to enter cytoplasmically.

In Fig. 1, the label has been changed to “Cavity site,” and the arrow has been extended to the central cavity. The last sentence of the legend has been adjusted accordingly to indicate that drugs that bind solely at the fenestration site or cavity site are listed in black, and the drugs that bind from both sites are listed in red. The corrected version of Fig. 1 and its legend appear below.

In the “Three disctint mechanisms of the pore blockers” section, the results of the simulations by Tao and Corry were misrepesented; they found that the drugs occupying the pore of the channel did not necessarily mean that they entered from the cytoplasm. In the last paragraph of the commentary, changes were made to accurately describe the three different modes of binding: “cavity block,” where the drug binds in the center of the pore, “fenestration block,” where it binds in the lateral fenestrations, and “versatile block,” where the drug can occupy either location.

The errors appear in PDFs downloaded before January 9, 2025.

**Figure 1.**
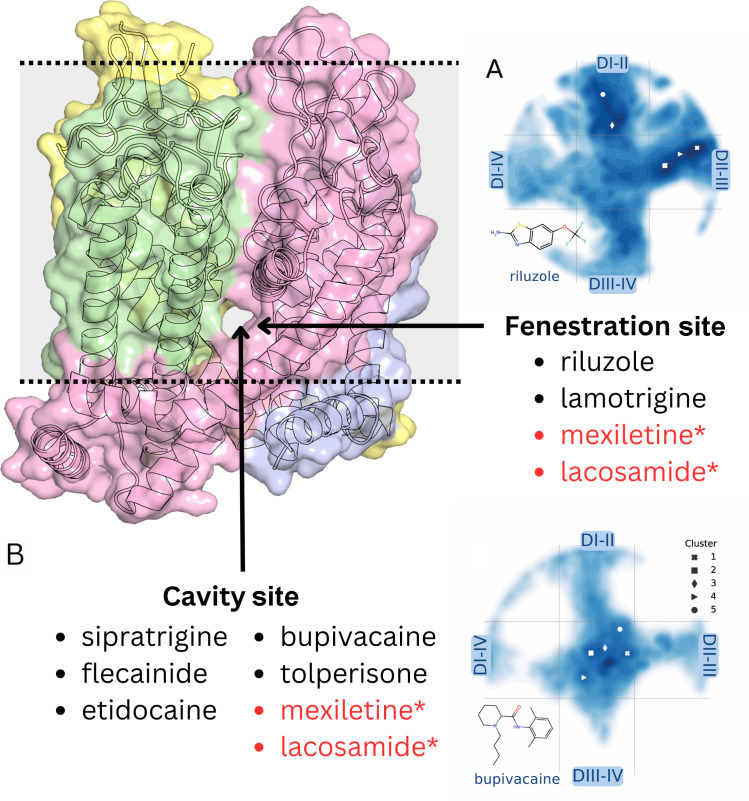
**Location of the binding sites of rat Na_v_1.5 channel pore blockers through the cavity site and/or the fenestration site.** The structure of the pore module of rat Na_v_1.5 (left) is shown as a surface. **(A and B)** Free-energy surface extract from Tao and Corry (2025) from metadynamics sampling using riluzole (A) or bupivacaine (B). Drugs that bind solely at the fenestration site or the cavity site are listed in black, whereas the drugs that can bind from both sites are denoted in red.
